# Civic communicators' view of and approach to health promotion for newly arrived migrants in Sweden

**DOI:** 10.3389/fpubh.2022.931685

**Published:** 2022-07-25

**Authors:** Sara Svanholm, Heidi Carlerby, Eija Viitasara

**Affiliations:** Department of Health Sciences, Mid Sweden University, Sundsvall, Sweden

**Keywords:** migrants, health promotion, civic orientation, empowerment, health education

## Abstract

For newly arrived migrants, integration is important in promoting health and decreasing health inequities. In a Swedish context, civic orientation is a program to promote integration and increase the chance of employment for newly arrived migrants. The aim of this project was to explore how civic communicators view and approach health promotion in their work with newly arrived migrants in the civic orientation program in Sweden. Data were collected through interviews with eight civic communicators working with newly arrived migrants in civic orientation in the north of Sweden. The interviews followed a semi-structured interview guide and were transcribed verbatim and analyzed using thematic analysis. The analysis resulted in the main theme “To dress the participants for a (healthy) life in Sweden,” with two sub-themes “Knowledge—a key to health” and “Being a guide for participants in a new context.” In their work with civic orientation for newly arrived migrants, civic communicators are involved in health promotion by preparing their participants for a life in Sweden. They work to empower their participants to be able to make informed decisions and live healthy lives by both providing information to enhance knowledge and skills. They also work to guide them through the complexity of being in a new situation and country.

## Introduction

Previous research has pointed out that migrants are at an increased risk of ill health in Europe and Sweden (1, 2). For example, so-called non-western migrants, largely refugees, and people who arrived through family reunification, have higher health risks and worse health outcomes as compared to natives and so-called western migrants, largely labor-related migrants, in Sweden (3). Syrians who have resettled in Sweden have a higher prevalence of mental ill health (4), as well as lower health-related quality of life (5) than the general population. Research on migrants in Finland shows that they describe having limited knowledge about healthy lifestyles and that they report having received limited information from the healthcare services regarding a healthy lifestyle (6). In a Swedish context, research has shown that newly arrived migrants have limited health literacy and ask for more information on health-related issues, as well as that the healthcare services sometimes take it for granted that migrants have the same general health knowledge as people who have grown up in Sweden (7). In regard to health literacy, or the knowledge, motivation and, competencies to access, understand, appraise and apply information relevant to health (8), migrants have been reported to have limited levels of health literacy both in Europe (9, 10) and Sweden (11, 12).

Migrant integration policies are important in the work of battling health inequalities (13). Integration can be defined as “*the degree to which immigrants have the knowledge and capacity to build a successful, fulfilling life in the host society*” (14). Post-migration factors, such as generous support from destination countries, are positively associated with better mental health for refugees (15). Time in the country of destination has shown to be important for health risks both in Sweden and Norway (3, 16). A study from Norway showed that individuals who had been in Norway a long time but had poor Norwegian proficiency had higher health risks than other migrants and native-born individuals (17). Health promotion, or “the process of enabling people to increase control over, and to improve, their health,” (18) has previously been pointed to as important in working with migrants (19), so also for newly arrived migrants in Sweden (5).

During the last decade, the number of migrants reaching Sweden has fluctuated. The number of asylum seekers went from ~30,000 a year at the beginning of the 2010s to a peak of 162,000 in 2015 when many arrived in Sweden due to the conflict in Syria (20) and then fell to ~13,000 per year at the beginning of the 2020s (21). Asylum seekers who have received residence permits, together with people who have arrived in Sweden through family reunification with former asylum seekers or people in need of subsidiary protection, the so-called newly arrived migrants, have the right to participate in an Establishment program. The Establishment program is a national program aimed at increasing newly arrived migrants' chances of entering the labor market and actively participating in society. One part of the program is the Civic Orientation (CO), in which they receive information about Swedish society (22).

The CO contains various information on topics such as living in Sweden, providing for oneself in Sweden, living with a family and children in Sweden, and caring for one's health in Sweden. The focus is meant to be on the practical aspects of everyday living in Sweden, and the CO should be adjusted according to local prerequisites. It was initially a 60-h program, but in 2020, the Government added 40 additional hours. The program is financed by the state, but it is the municipalities that are responsible for planning and delivering it. If a municipality chooses to commission someone else to plan and deliver the program, the municipality is still responsible for approving the plan (23).

The CO has been the focus of research previously, for example, from the participants' perspective, showing both the need for health information (7) and the fact that this is a valuable course for newly arrived migrants (24). Other studies have focused on sexual and reproductive health (25, 26) and health literacy (8). Through semi-structured interviews with civic communicators in municipalities in the north of Sweden, the aim of the current study was to explore how civic communicators view and approach health promotion in their work with newly arrived migrants in the civic orientation program in Sweden.

## Materials and methods

A qualitative study was designed to explore the civic communicators' view of and approach to health promotion. To capture the civic communicators' experiences and thoughts, interviews were chosen for data collection (27). To interpret the data, thematic analysis was chosen (28).

### Data collection

Information about the study and an invitation to participate was e-mailed to the contact person for the CO in each municipality. There was a total of fifteen such municipalities, all in two regions in the north of Sweden. The contact person was asked to forward the invitation e-mail to all communicators working with the CO in their municipality or e-mail their contact information so the researcher could send the invitation directly. At this stage, four municipalities replied that they did not currently have CO courses, due to outsourcing such to other municipalities or private actors. The remaining municipalities were all invited to participate, and they were reminded about the study once. Eight civic communicators agreed to participate in the study, and interviews over Zoom, over the phone or, in person, depending on their choice and convenience due to COVID-19 as well as the distance to the interview site, were scheduled.

All interviews were conducted in Swedish, five over Zoom, one over the phone, and two in person, and all followed a semi-structured interview guide (see Supplemental Material 1). After permission was obtained from the informants, the audio was recorded. The interviews were performed by the first author and ranged from 35 to 44 min. Following the interviews, the participants were offered a chance to read the transcripts of the interviews, and they could raise questions or concerns if something had been misunderstood. Five of the informants wanted to receive the transcripts, and none raised objections to the material.

The data collection took place in November 2021, which was during the COVID-19 pandemic, but during a time when the local transition was low, and activities were back to nearly normal. The classes were in person, and all communicators worked on-site. The only recommendations in place were to have larger classrooms with longer distances between students and that people with symptoms of COVID-19 had to stay home from classes.

### Participants

The informants were between 41 and 68 years old. Half were men, and their experience in their occupation ranged from 4 to 13 years. Five of the informants had personal experience of international migration. The education levels of the informants varied. One had a Swedish university degree, and one had a university degree from their country of origin and a health and civic communication education. Three other informants had the same health and civic communication education, and one of those was also an assistant nurse. Another informant was also an assistant nurse but without health and civic communication education, and two had completed shorter courses provided by their employer, for example, focused on pedagogy or communication.

### Analysis

A thematic analysis was performed to identify, analyze, and describe patterns and themes in the data (29). The recorded interviews were transcribed verbatim and checked against the audio to ensure accuracy (29). The transcribed data were imported to NVivo, which was used for the coding process. In line with Braun and Clarke's (29) description of thematic analysis, the data were read through, and the material was coded with words and phrases that described the content. The codes were then, based on their content and similarities, compared with one another, and collected into potential themes. Comparisons between the codes, between and within the themes, and the excerpts of the transcriptions were carried out throughout the entire analysis. Discussions about the themes and their content were also carried out in the research group throughout the analysis. A thematic map and, later, a table were used to finalize the thematic analysis before writing it up. The descriptions of the themes included detailed descriptions and vivid excerpts from the data (29). Quotes from the data were chosen so as to illustrate the themes and also represent all included informants. The quotes were translated from Swedish into English by the first author.

### Ethics

The Research Ethics Committee of Mid Sweden University reviewed the study (MIUN 2021/6) and raised no objections from an ethical point of view. In line with good research practice (30), the informants received written information and an invitation to participate in the study. Before the interviews began, they received verbal information about the study, the intention to publish, the fact that their participation was voluntary, the fact that they could leave the interview at any time without stating a reason, and the fact that their participation would remain confidential. They signed a written consent form before the interview began. For the interviews conducted over zoom or phone, the participants scanned or photographed the signed consent form and emailed it to the interviewer, and the participants who were interviewed in person signed and handed in a physical copy.

## Results

The data from eight civic communicators (informants) showed variation in how they view and approach health promotion within the program, but they all expressed that they thought health was an important subject to work within the CO for newly arrived migrants (participants). The analysis of the data resulted in one main theme: *to dress the participants for a (healthy) life in Sweden*, with two sub-themes, *Knowledge – a key to health* and *Being a guide for participants in a new context* (see Table 1).

**Table 1:** Illustration of theme and sub-themes.

**Theme**	**To dress the participants for a (healthy) life in Sweden**	
Sub-themes	Knowledge – a key to health	Being a guide for participants in a new context
Important aspects of the sub-themes	Lack of knowledge increases ill health	Support in learning and practical actions
	Receiving information improves health	Notice signs of ill health
	Health-related information	Help with contacts and practical aspects of health
	Information about society	Professional vs. private roles
	Theoretical and practical information	Flexible way of working
	Being the bringer of information	

### To dress the participants for a (healthy) life in Sweden

The main theme visible in the interviews, “*To dress the participants for a (healthy) life in Sweden*,” was focused on the informants' descriptions of how they worked to prepare their participants for life in Sweden. There was a duality in terms of how they described preparing the participants. The informants described this both in regard to the general information about Swedish society, which would prepare the participants for dealing with everyday life, and also specific health-related factors. The informants stressed that it was their role to prepare the participants so that they could make informed decisions and live healthy lives.

“*It all boils down to getting knowledge and the prerequisites to be able to make an active, informed, choice. If I do A, this happens, and if I do B, that happens” (Informant 6)*.

Through the interviews, the informants stressed the participants' individual responsibility to make healthy choices and have healthy habits. They could work with the participants to create an as good starting point for their lives in Sweden as possible. The more direct kind of help and support was described as a short-term solution while increasing the participants' skills and knowledge was the long-term solution.

The informants worked to create a common understanding of being in a new context with their participants. The informants who had personal experience of migration described that they used themselves as an example. The informants who did not have personal experience described how they imagined being new in their participants' country of origin so as to create an understanding of the situation and then worked from that point of view. They all described how they approached their work, both the component that was directly health related and the others, by viewing obtaining knowledge as a key to success, as well as that their role in guiding the participants through the CO was crucial to the participants' chances for successful, healthy lives in Sweden.

### Knowledge—A key to health

Throughout the interviews, the informants repeatedly returned to how important knowledge and information are. The informants described their view of their participants' needs from two main points of view: the new context (the Swedish society) and how this created a need for a new kind of information among the participants, as well as how the participants sometimes, because of their background, lacked a certain kind of information. Examples of a kind of information that many participants lacked include information about the female body, menstruation cycles, or mental health issues. Not having all the information or knowledge needed was often connected to ill health, especially by causing stress for the participants.

“*That's why I explain about the Swedish Migration Agency. Some people […], they get stuck in the process. There are steps left […]. They just wait and wait. And, then, the Migration Agency says they must wait 6 months and, then, 8 months, and they haven't been informed [about the process]. Then, it leads to ill health. They can't sleep. They feel bad” (Informant 4)*.

Receiving information was viewed not only as preventing ill health but as promoting good health. In response to a direct question about whether and how the CO could promote the participants' health, many of the informants stated that this was possible because of the information they gave the participants.

“*Yes, through the information. I can [affect health] through the information” (Informant 1)*.

The informants described the kind of knowledge needed by the participants from two points of view. In part, they stated that the participants needed more health-related knowledge. The most obvious type of information, which was often spoken about, is information regarding healthy living habits. The informants explained that they talked about how a person eats healthy and how physical activity is important with their participants. They further described that many participants do not have the contextual knowledge of, for example, what Swedish food could be considered healthy in regard to nutrients. They also described that even understanding what different kinds of foods were or how they were used was difficult for many participants given new packaging and new kinds of food items in stores.

“*To promote health, we can [talk about] what food is better for health, that walking is better for health, to know which food you should eat, [if it is] traditional and cultural foods aren't important, to know protein, what has sugar and salt, that affects health.” (Informant 7)*.

The informants described that it was not only through health-related information the participants' health was promoted but also through all the other information about the Swedish society they received.

“*This is my view, but if you understand the system and you know how things work and have read about it and all those things, then when stuff happens, when there's a problem, where am I gonna turn? You need to know that. You need to understand the system; then, you're not getting stressed” (Informant 4)*.

Apart from directly health-related information, the informants also explicitly pointed out the other information they provided, such as information about Swedish society, the labor market, and the various rights and obligations the participants have. The informants stressed the connection between a good integration, that is, having a job and an income, and good health.

“*Some kind of employment, not just getting financial aid, so that they get out. Something that… fills a function, doing something. Getting out of the home in the morning, having the kids at daycare. They get social and learn the language and learn everything. That's what the society can do [to promote health]” (Informant 6)*.

It was not only theoretical information that the informants described as important for promoting their participants' health. They also pointed out the chances and necessity of introducing the participants to the local context. They described how they plan active, or practical, lessons themselves, as well as visits to important local areas. Practical lessons could involve attempting various physical activities or going grocery shopping. Study visits were often paid to local health centers, where the participants could meet people working in various health professions. The informants pointed out how, later, it would be important for the participants to have both the knowledge and courage to return to healthcare services.

“*There are many professions: counselors, psychologists, nurses, doctors. They help each other as a team. When we come for a visit, they all say ‘welcome' and describe what they do. That's better than what I can say about the healthcare center. In a visit, they can see, and a doctor can describe [their work], or a nurse or a counselor [can do so]. That's better. I believe that. It feels like it” (Informant 7)*.

Several of the informants described that they used to have more time for health-related activities, such as physical activities or visiting local sports teams, attempting various sports, or having breaks to take walks together. This was possible because the local municipalities had put aside extra hours for health-related activities, beyond the then-mandated 60 h of CO. This had changed during the last years, however, the informants described, that new content directives and 40 more hours had been dictated, and financed by the state, which had “eaten up” the health-related hours. Instead of making this 140 h, the informants described that it was still 100 h, it was just the financing that had changed, and more content had been added. This caused a problem in terms of prioritizing health, which many of the informants stressed as important because many of the other included aspects were also considered important for the participants to take part in. They described a need for more time for the CO, with so many important aspects to cover within the program.

“*These 100 h are including those 40 that the Government added to include human rights, violence against women and, domestic violence, so we have a lack of time now. Very much. That means we must focus on the theoretical instead of the practical. We must. Before we had 100 h, which means 60 h CO and 20 added hours for health and 20 h for parental support education, but now, with these extra 40 h from the Government, it means 100 h, not 140 h” (Informant 8)*.

### Being a guide for participants in a new context

The second sub-theme, “*Being a guide for participants in a new context,”* is focused on how the participants viewed and described their role in how they work within the CO. It became clear that the informants guided the participants through the theoretical and practical aspects of education and being new in Sweden. The informants described that they support and help their participants in navigating their “new life.” An often-used example is contact with the healthcare system, which is described as difficult to deal with for newcomers in Sweden, especially for people who do not speak Swedish or English.

“*We guide them to where they can get help or support with this [health] problem. We can contribute so they end up at the right place and can get help” (Informant 5)*.

Related to the previous sub-theme focused on knowledge or information, the informants stressed that one of their important roles was being a bringer of knowledge. This was true both regarding theoretical knowledge and experience of living in a Swedish context. The informants were treated as experts in a wide variety of fields, and they often advised their participants.

“*If it's like you have problems sleeping for example, maybe you can do these things. Or, if you think the winter is boring when it's a lot of snow and you don't exercise anymore and are just inside, then you can get studded shoes and try to walk outside anyway. Yeah, I think that's important” (Informant 3)*.

Apart from relaying information, the informants also described that their role was to support the participants on a more individual level, both in their learning but also in dealing with specific problems, such as ill health. This was described as a short-term solution.

“*Those with mental health issues, we can try to support them, of course. We can help them with so many things, even with some of these mental health problems. I don't know… You need to find a solution to every problem. That is if it isn't a disease. We can help with things that have to do with a job, living situation, or children and family as well. Many things are fixable” (Informant 2)*.

Another way in which the informants described that they helped their participants was through noticing and picking up on symptoms of ill health, especially early signs of mental ill health. They described that, if they did not notice these things, no one might see it.

“*I can see it in my meetings with my participants. You see it on another human. You notice if they're not… in synch with their health. And, then, I think it's important to point it out” (Informant 3)*.

Through the interviews, the informants described practical ways in which they helped their participants. This was often related to contacting healthcare professionals but also things like talking with landlords and translating letters.

“*I care about their health. I don't just want them to get a job. I want them to feel well, and I can be the first person to answer these questions [about health]” (Informant 3)*.

One difference between the informants was how they described being an inspiration for their participants. It was the informants who had personal experiences of migration who described being an inspiration for the participants as an important part of their work.

“*And I talk about my experiences. I ask about experiences, and I talk about Sweden, and I talk about how you… I don't know the word… how to be accepted into society, to understand the society. All this makes you feel better, that you evolve and move on in the society” (Informant 2)*.

Sometimes, the distinction between the professional role as a civic communicator and what they helped their participants with outside of their role was not a clear one. This was especially true for the informants who had personal experience of migration and spoke the same language as their participants. The informants became a resource for the participants. This was especially true regarding sensitive subjects such as ill health or violence.

“*After class, they call and want guidance” (Informant 1)*.

The informants continuously described that they were on their participants' side. Other authorities were described as almost working against them sometimes and following rigid systems in which, for example, the digitalization of their way of working was described as harmful to the participants. The informants did not reflect on the municipality as an authority or how that affected their participants' views of them. Many described the municipalities' responsibility for their inhabitants and how this stood in contrast to other authorities that would, for example, cause financial troubles for the participants because they could not fill out the right information in an online system. The informants described a more flexible way of working, in which they wanted to solve problems created in other parts of the integration process.

“*The system is so complex. The system has high demands, but they don't reach it. How can we balance it? We need to make it simpler” (Informant 4)*.

## Discussion

The aim of the present study was to explore how civic communicators view and approach health promotion in their work with newly arrived migrants in the CO program. An analysis of semi-structured interviews with eight civic communicators working with CO within the Establishment program in Sweden showed that the civic communicators viewed and approached health promotion as an opportunity *to dress the participants for a (healthy) life in Sweden*. The sub-themes identified were *Knowledge – a key to health* and *Being a guide for participants in a new context*.

The main theme of the results—to dress the participants for a (healthy) life in Sweden—showed how the informants described how they worked to prepare their participants for a life in Sweden. They approached this through a focus on what kind of information the participants needed but also other skills and support that the informants guided their participants through. A focus was on ensuring the participants would be able to make decisions about their own lives, decisions that would affect their health positively. This is not only health promotion in its purest form, with a focus on enabling people to improve their own health (18), but also what is needed for integration (14). The informants stressed the fact that almost everything included in the CO promoted participants' health. A previous study showed that, although the Establishment program includes several aspects that promote health, it has not, in official policies, been defined as a health promotion initiative (31). In the present study, it was clear that the informants understood their role and chance to promote health, both in their individual actions as civic communicators and through the CO's role.

From the informants' descriptions, it was clear that their work is in line with empowerment as a concept. Empowerment can be viewed as either a goal or a process. It can be defined as working to support an individual or group in gaining control over the determinants of their lives through an increase in knowledge, health, or freedom. As well as minimizing the power gap between the “teacher” and the individual or group's goal and emphasizing decision-making, acting, and increasing their control over the process (32). Empowerment was a central aspect of how the informants described civic orientation. The informants described that they found it important that the participants, via their participation in the CO, received information and strategies for their future life. They built a toolbox and obtained all the prerequisites necessary to take responsibility for their own development, health-related and otherwise, in life. This is in line with how Tengland (32) states that both knowledge and skills are needed to truly enhance empowerment.

Interestingly, the presence of an empowerment perspective in the CO stands in contrast to how previous research has described that, in policies related to migration, the empowerment perspective is often lacking, and migrants are described as passive objects (33). This could explain why civic communicators do not expressly use the word “empowerment.” It is not an expressed strategy pointed out in policy but, instead, simply how the civic communicators choose to work. This opens up room for differences in strategies between civic communicators and, therefore, risks creating inequalities in the CO the participants receive. However, a previous study showed that participants reported that independence and self-confidence were increased by participation in CO (24).

One problem within health promotion for migrants that has been reported previously is the lack of cultural sensitivity within the programs (19). This was not something that the informants in the current study discussed outright, but they often returned to the importance of forming a common understanding of the context. Previous research has reported that healthcare personnel with a migration background have reported their background as an important factor that increased migrants' trust in them (34). Informants in the current study who shared cultural background or experience of migration with their participants described that they used this to level the playing field and approach the information within the CO together. The informants without such experience used other strategies, such as imagining themselves as migrants, to create the common ground with their participants. This form of health promotion, which is performed without health professionals, could be considered closer to peer education, which is an effective method of promoting health for migrants (19), especially using bi-lingual workers who share a cultural background with the participants (35). However, to be certain that cultural sensitivity within the program was not a problem, the perspective of the participants would be needed.

In the current study, the informants described that being an inspiration, that is, a positive role model, was important as a civic communicator. This was specifically described by the informants who shared a migration background with their participants. This is in line with previous research in which Al-Adhami et al. (24) describes that participants report being inspired to focus on their health after participating in CO.

Throughout the interviews, the informants stress how important knowledge is for the participants in being able to take control over their lives in Sweden. They point out how many participants have insufficient knowledge about how their body works, what healthy habits look like, and how different aspects of Swedish society works. This is in line with a previous Swedish study that has shown that migrants “lack knowledge” about certain health-related issues and also about the health system in Sweden (7). A study in a Finish setting also showed that migrants experience they have limited knowledge of health-related issues and that this, together with miscommunication, can create problems in the contact between migrants and healthcare personnel (6). Both knowledge and skills, which were the important aspects of how the civic communicators approached health promotion, are also important aspects in regard to health literacy (36). It answers the issue previously raised when research has shown that newly arrived migrants point out the need for information (7). Newly arrived migrants have also pointed out that they consider the CO important and valuable (24).

However, another important aspect is the shift from individuals with the civic orientation, to the society at large. The informants in the current study stressed not only how important it was to ensure that their participants have access to all information they need but also how other institutions in society had to adapt their way of working to suit the entire population. They mentioned the Public Employment Agency's digitalization and the healthcare services' use of only Swedish as two main problems for many migrants in Sweden today. They stressed that, even though their participants learned to deal with society, it was also society's responsibility to accommodate the entire population. This is something that has also been raised from newly arrived migrants' perspective in previous health literacy research, that an adaption in for example the healthcare services is needed to accommodate their needs (7). Further, it is connected to how important the structural factors and social determinants of health are, as well as how they affect the entire population's health (37, 38).

The new knowledge on how civic communicators work with health promotion, from the current study, adds an important aspect for further research and policy implications. Especially in addition to how newly arrived migrants have reported that the civic orientation is valuable (24), but also to the fact that societal change is needed (7). Since neither health promotion (31), nor empowerment (33), are perspectives within integration policies, there is a need to strengthen this perspective within the policy process regarding the integration of newly arrived migrants in Sweden.

### Methodological considerations

To ensure a rigorous analysis, the 15-point checklist of criteria for good thematic analysis (29) was used. To ensure trustworthiness, the credibility, transferability, dependability, and confirmability of the study were considered throughout every step of the study (39). To ensure credibility, that is, that the study actually studies what it intends (40), civic communicators currently working with the CO were chosen as informants. All civic communicators working in the two selected regions were invited so as to minimize selection bias, and eight chose to participate. In the final few interviews, similarities in the subjects brought up by the informants were visible, and data saturation was reached. Transferability cannot be provided or promised by the researchers but is, rather, up to the readers of the study (40). However, to increase the transferability of the results, both the participants and the context of the study were described in detail (41). Also, the method has been outlined in detail to ensure transparency in the process leading up to the results, as well as ensuring dependability (40).

For reflexivity, the research team's pre-understanding is important (42). The first (SS) and second authors (HC) have experience working with health education for civic communicators and have developed, through this work, a deeper understanding of the context in which civic communicators work. Four of the civic communicators included in the study have taken a course that the researchers have participated in the design of. The researchers and informants had never met, and the researchers had not been personally involved with the informants' education, only in the creation of the course. This pre-understanding has been considered throughout the study. This was mainly done through discussions within the research team. A semi-structured interview guide was created to ensure that the interviewer (SS) collected the informants' experiences and words and was not steered by pre-understanding. During the coding and forming of themes, all three researchers discussed the themes, the risk of being influenced by pre-understanding, and the experience of the interviews to ensure the themes were derived from the data. The handling of pre-understanding is important for the study's trustworthiness through confirmability (41). However, pre-understanding could also be considered a strength in that the researchers understood the policy context and the organization around the civic communicators, which was important during the design of the study, and thus increased credibility (40).

## Conclusion

This study shows that civic communicators, in their work with civic orientation for newly arrived migrants, work with health promotion by preparing their participants for a life in Sweden. They work to empower their participants to be able to make informed decisions and live healthy lives by providing information to enhance knowledge and skills and also by guiding them through the complexity of being in a new situation and country.

## Data availability statement

The original contributions presented in the study are included in the article/supplementary material, further inquiries can be directed to the corresponding author/s.

## Ethics statement

The studies involving human participants were reviewed and approved by the Research Ethics Committee of Mid Sweden University. The patients/participants provided their written informed consent to participate in this study.

## Author contributions

SS performed material preparation, data collection, analysis, and wrote the first draft of the manuscript. All authors contributed to the study conception and design, commented on previous versions of the manuscript, read, and approved the final manuscript.

## Funding

The open access publication fee is covered by Department of Health Sciences, Mid Sweden University.

## Conflict of interest

The authors declare that the research was conducted in the absence of any commercial or financial relationships that could be construed as a potential conflict of interest.

## Publisher's note

All claims expressed in this article are solely those of the authors and do not necessarily represent those of their affiliated organizations, or those of the publisher, the editors and the reviewers. Any product that may be evaluated in this article, or claim that may be made by its manufacturer, is not guaranteed or endorsed by the publisher.
