# Supplementary material for: Civic communicators' view of and approach to health promotion for newly arrived migrants in Sweden
Source: Front Public Health. 2022 Jul 25;10:931685. doi: 10.3389/fpubh.2022.931685 (PMC9357985; doi:10.3389/fpubh.2022.931685)
Supplement: Supplementary file 1 [file Data_Sheet_1.DOCX]

# Semi-structured interview guide

## Interview guide in English

Translated for publication.

### Introductory questions

How would you describe your work as a civic communicator?

Have you any experience working with health?

### Main questions

What does the concept of health mean to you?

What affects people’s health?

Is there a difference between newly arrived migrants compared to the rest of the population?

How do you work with health in Civic Orientation?

Do you think the Civic Orientation can affect newly arrived migrants’ health?

Can you/the civic communicator affect newly arrived migrants’ health?

How do you think newly arrived migrants’ health is in Sweden now?

Do you think there is a difference in health related to if newly arrived migrants are placed in a large or small municipality?

What would have to be done to improve newly arrived migrants’ health? What societal interventions can be done?

Who in society do you consider having the possibility to affect newly arrived migrants’ health?

Who in society do you consider having the responsibility to affect newly arrived migrants’ health?

### Concluding questions

Is there anything more related to newly arrived migrants’ health that you would like to share with me? Something that we have not touched upon in the interview.

Is it okay if I contact you later in the process if there are questions?

Is it okay if I contact you when I have transcribed this interview, so you can take part of the material?

### Background questions

Is the civic orientation in your municipality performed by physical meetings or digital meetings? Are you employed by the municipality?

Age?

Gender?

How long have you worked as a civic communicator?

Have you partaken in any education related to health?

Do you have personal experience with migration?

## Interview guide in Swedish (original language)

### Introduktionsfrågor

Hur skulle du beskriva ditt jobb som samhällskommunikatör?

Har du någon erfarenhet av att arbeta med hälsa?

### Huvuddelen av intervjun

Vad betyder begreppet hälsa för dig?

Vad påverkar människors hälsa?

Är det olika saker för nyanlända jämfört med övriga befolkningen?

Hur arbetar ni med hälsa i samhällsorienteringen?

Anser du att samhällsorienteringen bidrar till nyanländas hälsa?

Kan du/samhällskommunikatörer påverka nyanländas hälsa?

Hur är din uppfattning att hälsan är bland nyanlända i Sverige?

Uppfattar du det som att det skiljer i hälsa bland nyanlända beroende på mottagarkommunens storlek?

Vad tror du skulle behöva göras för att bidra till nyanländas hälsa? Vilka samhällsinsatser kan genomföras?

Vilka i samhället har möjlighet att bidra till nyanländas hälsa?

Vilka i samhället har ansvar för att bidra till nyanländas hälsa?

### Avslutande frågor

Är det något mer angående nyanländas hälsa som du skulle vilja dela med dig av, är det något som inte har kommit upp/berörts tidigare i intervjun?

Går det bra att jag kontaktar dig igen om det är någonting som är oklart i ett senare skede?

Går det bra att jag kontaktar dig igen efter jag transkriberat denna intervju, så får du möjlighet att kolla materialet innan vi går vidare med analysen?

### Bakgrundsfrågor

Samhällsorientering distans eller fysiskt? Kommunal eller privat utförare?

Ålder?

Kön?

Hur länge har du arbetar som samhällskommunikatör?

Har du gått någon utbildning som innehåller hälsa av något slag?

Har du egen erfarenhet av migration?
